# Genome-Wide DNA Methylation Changes between the Superficial and Deep Backfat Tissues of the Pig

**DOI:** 10.3390/ijms13067098

**Published:** 2012-06-08

**Authors:** Mingzhou Li, Tao Wang, Honglong Wu, Jie Zhang, Chaowei Zhou, Anan Jiang, Ruiqiang Li, Xuewei Li

**Affiliations:** 1 Institute of Animal Genetics & Breeding, College of Animal Science & Technology, Sichuan Agricultural University, Ya’an 625000, China; E-Mails: mingzhou.li@163.com (M.L.); wanttao@gmail.com (T.W.); zhangjie813@163.com (J.Z.); zcwlzq666@163.com (C.Z.); lingdang317@163.com (A.J.); 2 Peking-Tsinghua Center for Life Sciences, Biodynamic Optical Imaging Center & College of Life Sciences, Peking University, Beijing 100871, China; 3 BGI-Shenzhen, Shenzhen 518000, China; E-Mail: wuhonglong@genomics.cn

**Keywords:** pig, adipose, superficial backfat, deep backfat, DNA methylation

## Abstract

Adipose tissue is not only a storage organ involved in fuel metabolism, but also an endocrine organ involved in the regulation of insulin sensitivity, thermogenesis, immunity, and inflammation. There are anatomical, cellular, molecular and physiological differences among adipose tissues deposited in different body sites. However, current understanding of the intrinsic differences between the sub-compartments of the subcutaneous adipose tissue remains rudimentary. Here, we analyzed the genome-wide DNA methylation differences between the porcine superficial and deep backfat tissues using methylated DNA immunoprecipitation combined with high-throughput sequencing. We show that the genes with differentially methylated regions in their promoter are mainly involved in the processes of “lipid metabolism” and “regulation of immune-related cytokines”. Compared with the deep backfat tissue, the promoters of genes related to the ‘positive regulation of cytokine production’ were significantly hypermethylated in the superficial backfat tissue, which reflects the intrinsic functional and metabolic differences between the sub-compartments of the subcutaneous adipose tissue. This study provides epigenetic evidence for functionally relevant methylation differences between different layers of porcine backfat tissues.

## 1. Introduction

Adipose tissue plays an important role in energy homeostasis, not only in storing triglycerides [1], but also in the secretion of many different hormones (termed adipokines) that control feeding, thermogenesis, immunity and neuroendocrine function [2]. Adipose tissues from different body sites display distinct structural and functional properties and have disparate roles in pathology [3,4]. It is well known that visceral adipose tissues are functionally distinct from subcutaneous adipose tissues (SATs), and they have been found to be related to a series of obesity-related metabolic and cardiovascular diseases [5,6]. SAT, conventionally regarded as a homogenous compartment, is anatomically separated by a stromal fascia into superficial and deep SAT [7]. Currently, the intrinsic differences between the SAT sub-compartments are not well understood [8–10].

In general, rodents have been used as models to study human obesity. However, the numerous disparate results between rodents and humans have hindered the translation of discoveries in rodents into effective preventive or interventional therapies for human obesity and its comorbidities [11]. The pig (*Sus scrofa*) is now a rapidly emerging biomedical model for energy metabolism and obesity in humans because pigs are closely comparable to humans in size, anatomy, physiology, metabolism, and pathology [12]. A better understanding of the mechanisms of adipose tissue accumulation in pigs would also contribute to improved pork production efficiency.

The classic genetic approach has considered some forms of obesity to be an inherited disease caused by gene mutations and polymorphisms [13]. However, common DNA sequence variants inadequately explain the variability in fat mass among individuals [14]. Recently, there has been a greater appreciation of the role of epigenetic factors in complex diseases, especially DNA methylation, which is a heritable modification affecting gene expression without change in the DNA sequence itself [15].

Here, we present a genome-wide analysis of DNA methylation as well as gene expression in the porcine superficial backfat (sBF) and deep backfat (dBF) tissues. We identified differentially methylated regions (DMRs), and found that the genes with DMRs in their promoter are primarily involved in the processes of lipid metabolism and the regulation of immune-related cytokines. Our results suggest that the dBF possesses an independent metabolic function that may play a role in obesity-associated immune disorders.

## 2. Results and Discussion

### 2.1. Characteristics of Porcine sBF and dBF Tissues

**Figure 1:**
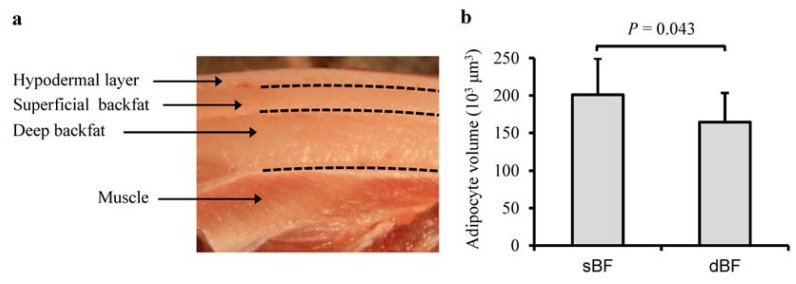
Phenotypic differences between the superficial backfat (sBF) and deep backfat (dBF) tissues. (**a**) Anatomical location; (**b**) Adipocyte volume difference. Student’s paired *t*-test (*n* = 9). Values are means ± SD.

The sBF and dBF tissues, which are located in different anatomical locations (Figure 1a), exhibited significantly different adipocyte volumes (*p* = 0.043) (Figure 1b); this confirms the findings of a previous report in which the adipocytes from deep sites were smaller than those from superficial sites [10]. In addition, the sBF and dBF tissues had distinct fatty acid compositions for saturated fatty acids (*p* = 0.005) and monounsaturated fatty acids (*p* = 0.038) (Table 1), which suggests that their metabolic activities, such as the rate of deposition and mobilization, and also the rate of endogenous synthesis of fatty acids, differ between the different body sites [16]. These phenotypic differences between the sBF and dBF imply intrinsic differences in their molecular regulation.

### 2.2. Characterization of DMRs

**Figure 2:**
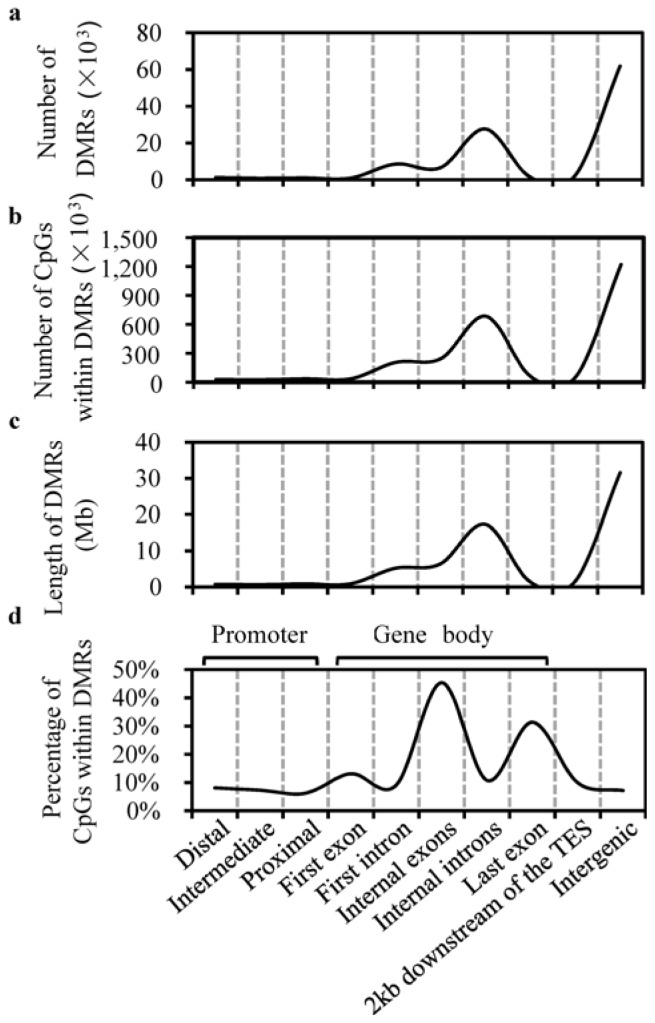
Distribution of differentially methylated regions (DMRs). (**a**) Number of DMRs; (**b**) Number of CpGs within the DMRs; (**c**) Length of DMRs; (**d**) Percentage of CpGs within the DMRs (CpG number in DMRs *vs*. the total CpG number in each genomic feature). The canonical gene structure was defined by 10 different features, denoted on the *x*-axis. TES: transcription end site.

We generated 42.80 gigabases (Gb) of methylated DNA immunoprecipitation sequencing (MeDIP-seq) data from six samples (~7.13 Gb per sample), of which 32.91 Gb (76.89%) of clean reads could be aligned on the pig reference genome. We used statistics to measure the methylation rate changes, and defined DMRs between the sBF and dBF tissues. The high correlation (average Pearson’s *r* = 0.995) between the number of DMRs (Figure 2a), the number of CpGs within the DMRs (Figure 2b), and the length of the DMRs (Figure 2c) implied that DMR detection in regions of different length and number of CpGs was non-biased. The number of DMRs varied considerably among 10 defined features of canonical gene structure: the distal (1254 DMRs), intermediate (877 DMRs), and proximal (1020 DMRs) regions of the promoter, the first exon (931 DMRs), the first intron (8518 DMRs), internal exons (6547 DMRs), internal introns (27,615 DMRs), the last exon (1429 DMRs), 2 kb downstream of the transcription end site (TES) (2356 DMRs), and intergenic regions (61,807 DMRs). We then looked at the percentage of CpGs within the DMRs for each genomic feature (Figure 2d). Intriguingly, DMRs occurred more frequently in exons (29.96%) than in promoters (% of CpGs within the DMRs, 7.22%), introns (9.95%), 2 kb downstream of the TES (9.97%), or intergenic regions (7.18%). A previous report indicated that 16% of all CpG islands in the human brain were methylated, whereas 98% of CpG islands associated with annotated promoters were unmethylated [17]. This suggests that in the human brain, DNA methylation may serve a broader role in intragenic regions than in promoters. DNA methylation in promoters suppresses gene expression, yet in the main body of the gene (particularly in coding exons), it is also important in regulating alternative promoters and preventing spurious transcription initiation [18].

### 2.3. Correlation between mRNA Expression and Methylation in Promoters

We explored the correlation between the changes of DMR in promoters and the associated mRNAs expression levels. To obtain high-confidence gene expression data, we mapped 43,603 60-mer probes of a microarray to the pig reference genome; this resulted in 4983 (11.43%) probes uniquely mapped to exons of Ensembl genes. Multiple probes that mapped to the same or different exons of a specific gene were filtered out. Only 3074 probes (7.05%), uniquely representing 3074 genes, were used in subsequent analyses.

**Figure 3:**
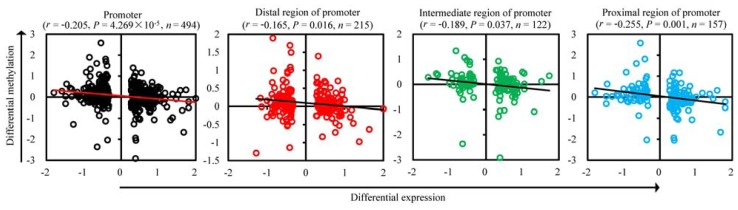
Correlation between mRNA expression and methylation in promoter sub-regions. The scatter plots and trend lines show the Pearson’s correlation between the log_2_ ratio of mRNA expression difference and the log_2_ ratio of the methylation difference in the whole promoter, and the distal, intermediate, and proximal sub-regions of the promoter. The line represents linear regression.

As shown in Figure 3, there was a negative correlation between mRNA expression and DMRs across the entire promoter (Pearson’s *r* = −0.205, *p* = 4.269 × 10^−5^). In detail, the correlation coefficient in the different promoter regions decreased from the proximal (Pearson’s *r* = −0.255, *p* = 0.001) to the intermediate (Pearson’s *r* = −0.189, *p* = 0.037) to the distal (Pearson’s *r* = −0.165, *p* = 0.016) region. This validates previous findings in human melanoma cells [19]. Transcriptional repression driven by promoter methylation is inversely related to the distance between the promoter sub-regions and the transcription start site.

### 2.4. Functional Differences between sBF and dBF Tissues

**Figure 4:**
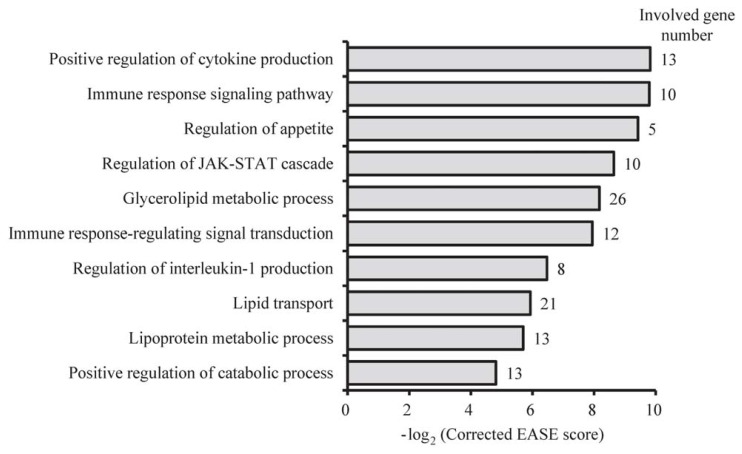
Top ten GO (Gene Ontology) categories enriched for genes with DMRs in the promoter. The EASE score, indicating the significance of the comparison, was calculated using the Benjamini-corrected modified Fisher’s exact test.

To identify genes potentially responsible for functional and metabolic differences between the sBF and dBF, we performed functional enrichment analysis of Gene Ontology (GO) for genes with DMRs in their promoters using DAVID software [20]. We found that the top 10 significantly overrepresented categories of GO biological processes (GO-BP) were related to the two main classes of biological functions (Figure 4), *i.e.*, ‘lipid metabolism’ (such as catabolic process, glycerolipid metabolic process, lipoprotein metabolic process, lipid transport, and regulation of appetite) and “regulation of immune-related cytokines” (such as cytokine production, immune response-regulating signal transduction, immune response signaling pathway, interleukin 1 production, and JAK-STAT cascade), which reflects the distinct roles of the sBF and dBF in energy homeostasis and adipokine-induced immune responses [21,22].

**Figure 5:**
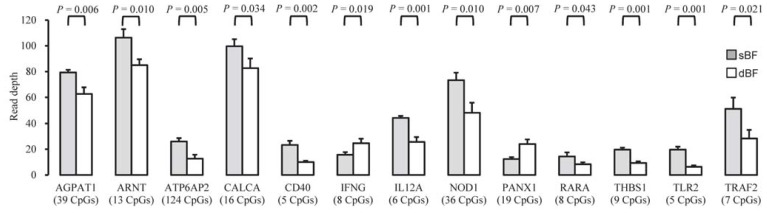
Differential DNA methylation in the promoters of 13 genes involved in positive regulation of cytokine production. Student’s paired *t*-test (*n* = 3). The number of CpG sites within DMRs are shown in parentheses. *AGPAT1*: 1-acylglycerol-3-phosphate *O*-acyltransferase 1; *ARNT*: aryl hydrocarbon receptor nuclear translocator; *ATP6AP2*:ATPase, H^+^ transporting, lysosomal accessory protein 2; *CALCA*: calcitonin-related polypeptide α; *CD40*: TNF receptor superfamily member 5; *IFNG*: interferon γ; *IL12A*: interleukin 12A; *NOD1*: nucleotide-binding oligomerization domain containing 1; *PANX1*: pannexin 1; *RARA*: retinoic acid receptor α; *THBS1*: thrombospondin 1; *TLR2*: Toll-like receptor 2; *TRAF2*: TNF receptor-associated factor 2.

Notably, the most significantly overrepresented category was enriched for 13 genes related to “positive regulation of cytokine production”, of which the promoters of 11 (84.62%) genes were significantly hypermethylated in the sBF compared with the dBF (Figure 5). This suggests a higher mRNA expression level of these genes in the dBF compared with the sBF, which may result in higher cytokine levels secreted from the dBF than from the sBF. It is well established that adipocyte-derived adipokines can regulate systemic processes, particularly those involved in insulin sensitivity, immune responses, and inflammation [21,23]. These results are consistent with those of a previous report, which found that, compared with human superficial SAT, deep SAT appears to be a distinct adipose depot that supports an independent metabolic function, and may be associated with the risk of obesity-associated complications [8]. It is believed that the SATs can have direct and beneficial effects on the control of body weight and metabolism [24]. Visceral adipose tissues were negatively associated with peripheral insulin sensitivity, while thigh SAT, in contrast, was positively associated with peripheral insulin sensitivity [25]. Our findings of anatomical location-specific methylation patterns imply intrinsic functional and metabolic differences between the superficial and deep SATs. A recent study of the autologous transplantation of visceral adipose tissues to subcutaneous sites demonstrated the impact of local (residence) factors influencing the epigenetic memory of adipose depots in different body sites [26].

## 3. Experimental Section

### 3.1. Animals and Tissue Collection

Nine healthy female Landrace pigs at 210 days old were used in this study. After sacrifice, the sBF and dBF tissues near the last third or fourth rib were rapidly separated from each carcass, immediately frozen in liquid nitrogen, and stored at −80 °C until RNA and DNA extraction.

### 3.2. Measurement of Adipocyte Volume

All adipose samples were fixed in 10% neutral buffered formalin solution, embedded in paraffin, sliced at a thickness of 6 μm using an RM2135 rotary microtome (Leica, Berlin, Germany) and stained with hematoxylin and eosin. The mean diameter of an adipocyte cell was calculated as the geometric average of the maximum and minimum diameter, and 100 cells were measured for each sample in randomly selected fields. The mean adipocyte volume (*V*) was obtained according to the following Formula 1:

(1) $$V=\pi /6\;\Sigma f_{i}{D_{i}}^{3}/\Sigma f_{i}$$

where *D_i_* is the mean diameter, and *f_i_* denotes number of cells with the mean diameter *D_i_*.

### 3.3. Measurement of Fatty Acid Composition

We determined the fatty acid composition of all adipose samples as previously described [27]. The fatty acid methyl esters (FAMEs) were quantified using GC-14C gas chromatography (Shimadzu, Kyoto, Japan). Response factors were determined by analyzing a standard solution of the relevant pure FAME. Individual compounds were identified by comparing their retention times with those of standards (Sigma-Aldrich, MO, USA). FAMEs were identified by comparison with standards previously run independently or together with samples.

### 3.4. MeDIP-Seq

We randomly selected three pigs as biological replicates. MeDIP DNA libraries were prepared following a previously described protocol [28]. In brief, DNA (5 μg) was sonicated to approximately 100 to 500-bp fragments with a Bioruptor sonicator (Diagenode, NJ, USA). Then, libraries were constructed using a Paired-End DNA Sample Prep kit (Illumina, CA, USA) following the manufacturer’s instructions. Adaptor-ligated DNA was immunoprecipitated by a monoclonal anti-methylcytidine antibody (Diagenode, NJ, USA). The enriched methylated fragments and 10% input DNA were purified on DNA Clean & Concentrator-5 columns (Zymo, CA, USA) following the manufacturer’s instructions. Enriched fragments were amplified by adaptor-mediated PCR. Each MeDIP library was subjected to paired-end sequencing with 50-bp read-lengths using an Illumina HiSeq 2000 Sequencing System. The MeDIP-seq data have been deposited in the NCBI Gene Expression Omnibus under the GEO Series accession number GSE30344.

### 3.5. Identification of DMRs

After filtering the low-quality reads, the MeDIP-seq data were aligned to the UCSC pig reference genome (Sscrofa9.2) using SOAP2 (version 2.21) [29]. The genomic regions differentially enriched in methylated CpGs between the sBF and dBF were identified using our newly developed method. First, the normality and equal variances of read depth at each CpG across different sample groups were tested using Bartlett’s test (passed for *p* > 0.05, failed for *p* < 0.05). Second, the parametric Student’s paired *t*-test (if the data passed the Bartlett’s test) or the non-parametric Wilcoxon rank-sum test (if the data failed the Bartlett’s test) were used to select the highly variable CpGs (*p* < 0.01) as the seed site of a candidate DMR. Third, the 3′ downstream adjacent CpGs were singly incorporated with this seed CpG. To highlight the CpG-enriched regions, we allowed up to 200-bp separation between two adjacent CpGs, with which ~88.8% of all CpGs in the pig genome were covered (Supplementary Figure S1). The average read depth of a defined multi-CpG region was repeatedly subjected to a new round of tests. The resulting *P* values for DMRs were corrected using the Benjamini-Hochberg method (false discovery rate < 0.01; 1,000 permutations). If five or more CpGs in a genomic region demonstrated a significantly different (*p* < 0.01) read depth between samples, this region was taken to be a DMR.

### 3.6. Definition of Genomic Elements

We identified the genomic locations of the promoters, exons, and introns of the 21,533 Ensembl genes, together with the 13,626 intergenic regions, by referring to the UCSC pig reference genome (Sscrofa9.2). Each promoter of 2700-bp length was divided into three regions as previously described [19]: proximal (−200 to +500 bp), intermediate (−200 to −1000 bp), and distal (−1000 to −2200 bp).

### 3.7. Gene Expression Microarray Analysis

Total RNA (10 μg) from three sBF and three dBF samples, which corresponded to the samples used for MeDIP-seq sequencing, was extracted with TRIzol (Invitrogen, CA, USA). The labeling procedure (Cy-3 dye only) was carried out using an RNA Fluorescent Linear Amplification Kit (Agilent Technologies, Santa Clara, CA, USA). The fragmented target was applied to a Pig Gene Expression Oligo Microarray (version 2; Agilent Technologies, Santa Clara, CA, USA, 2011). Data analysis was performed with MultiExperiment Viewer (MeV) [30]. The microarray data have been deposited in the NCBI Gene Expression Omnibus under the GEO Series accession number GSE30343.

## 4. Conclusions

In summary, we present epigenetic evidence for functionally relevant methylation differences between different layers of porcine backfat tissues. Through identification of DMRs and analysis of the relationship between changes in mRNA expression and methylation in promoters, we found that DMRs in promoters can repress gene expression. We also found that “lipid metabolism” and “regulation of immune-related cytokines” are the most significantly different functional categories between the sBF and dBF tissue, which reflects the intrinsic functional and metabolic distinctions between the sub-compartments of the SAT. These observations are a prelude to in-depth investigations of the causal direction of anatomical location-specific methylation patterns in obesity-derived immune dysfunction, and will also be useful in maximizing the production of high-quality pork.

## Supplementary Information



## Figures and Tables

**Table 1:** Fatty acid composition differences between the superficial backfat (sBF) and deep backfat (dBF) tissues.

Fatty Acids	sBF	dBF	*p* Value
**SFA**	35.63 ± 2.09	37.39 ± 2.19	0.005
**MUFA**	44.92 ± 0.80	43.54 ± 1.27	0.038
**PUFA**	19.45 ± 1.31	19.06 ± 0.97	0.235

SFA, MUFA and PUFA are saturated, monounsaturated, and polyunsaturated fatty acids, respectively. Values are means ± SD. Student’s paired *t*-test (*n* = 9).
