# Supplementary material for: Genome-Wide DNA Methylation Changes between the Superficial and Deep Backfat Tissues of the Pig
Source: Int J Mol Sci. 2012 Jun 8;13(6):7098–108. doi: 10.3390/ijms13067098 (PMC3397513; doi:10.3390/ijms13067098)

Genome-Wide DNA Methylation Changes between the Superficial and Deep Backfat Tissues of the Pig

Supplementary Information

Figure S1. Distribution of CpGs in pig genome against distance between adjacent CpGs (bp). The accumulative % (bar) and % (black line) of CpGs against adjacent distance are charted in 10 bp windows beside the first class (2–10 bp). The adjacent distance of 88.82% CpGs in pig genome is within 200 bp (dashed horizontal line and black bar).

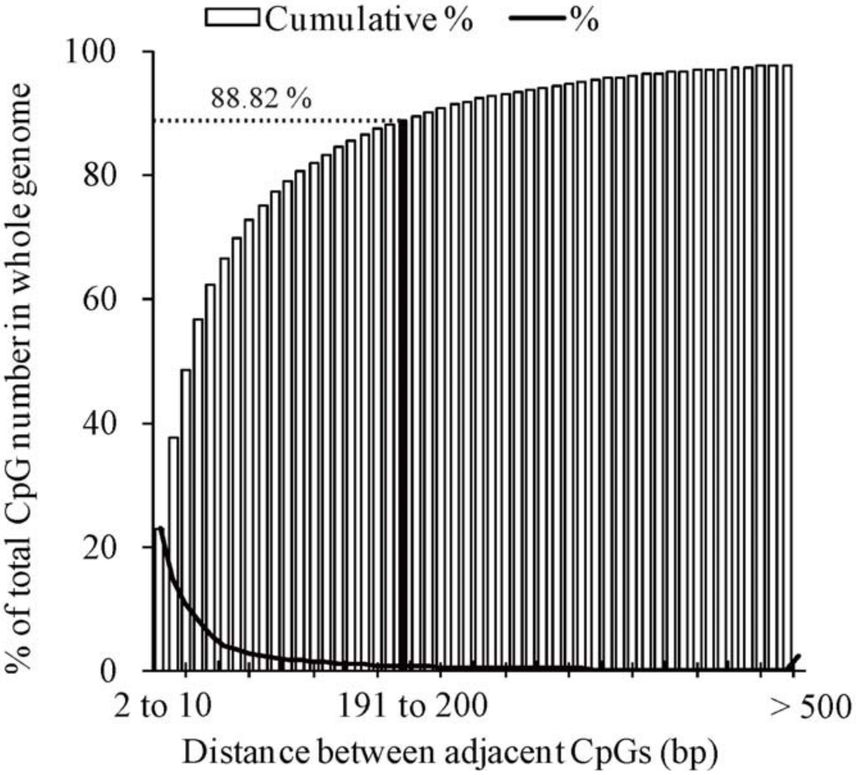

Supplement: Supplementary file 1 [file ijms-13-07098-s001.pdf]
